# Maturation profile of inferior olivary neurons expressing ionotropic glutamate receptors in rats: role in coding linear accelerations

**DOI:** 10.1007/s00429-012-0432-3

**Published:** 2012-06-16

**Authors:** Chuan Li, Lei Han, Chun-Wai Ma, Suk-King Lai, Chun-Hong Lai, Daisy Kwok Yan Shum, Ying-Shing Chan

**Affiliations:** 1 Department of Physiology, LKS Faculty of Medicine, The University of Hong Kong, 21 Sassoon Road, Hong Kong, People’s Republic of China; 2 Department of Biochemistry, LKS Faculty of Medicine, The University of Hong Kong, 21 Sassoon Road, Hong Kong, People’s Republic of China; 3 Research Centre of Heart, Brain, Hormone and Healthy Aging, LKS Faculty of Medicine, The University of Hong Kong, 21 Sassoon Road, Hong Kong, People’s Republic of China; 4 Present Address: Department of Medical Science, Tung Wah College, Wyile Road, Kowloon Hong Kong, People’s Republic of China

**Keywords:** Postnatal, Gravity, Inferior olive, Spatial map, Glutamate receptor, Vestibular system

## Abstract

Using sinusoidal oscillations of linear acceleration along both the horizontal and vertical planes to stimulate otolith organs in the inner ear, we charted the postnatal time at which responsive neurons in the rat inferior olive (IO) first showed Fos expression, an indicator of neuronal recruitment into the otolith circuit. Neurons in subnucleus dorsomedial cell column (DMCC) were activated by vertical stimulation as early as P9 and by horizontal (interaural) stimulation as early as P11. By P13, neurons in the *β* subnucleus of IO (IOβ) became responsive to horizontal stimulation along the interaural and antero-posterior directions. By P21, neurons in the rostral IOβ became also responsive to vertical stimulation, but those in the caudal IOβ remained responsive only to horizontal stimulation. Nearly all functionally activated neurons in DMCC and IOβ were immunopositive for the NR1 subunit of the NMDA receptor and the GluR2/3 subunit of the AMPA receptor. In situ hybridization studies further indicated abundant mRNA signals of the glutamate receptor subunits by the end of the second postnatal week. This is reinforced by whole-cell patch-clamp data in which glutamate receptor-mediated miniature excitatory postsynaptic currents of rostral IOβ neurons showed postnatal increase in amplitude, reaching the adult level by P14. Further, these neurons exhibited subthreshold oscillations in membrane potential as from P14. Taken together, our results support that ionotropic glutamate receptors in the IO enable postnatal coding of gravity-related information and that the rostral IOβ is the only IO subnucleus that encodes spatial orientations in 3-D.

## Introduction

Head movement signals, detected by vestibular end organs, are processed in the vestibular nuclei and cerebellum to elicit stabilization of posture and eyes. Vestibular nuclei and cerebellum are intimately connected in a reciprocal manner. Outputs from the vestibular nuclei are transmitted to the cerebellum either directly to the granule cells or indirectly via the inferior olive (IO) (Gerrits et al. 1985) which is indeed the sole origin of climbing fibres to Purkinje cells of the cerebellum (Flumerfelt and Hrycyshyn 1985). Upon integrating the former input from granule cell-parallel fibre and the latter from olivo-cerebellar projection, Purkinje cells in turn send axons to the vestibular nuclei (Ito 2002). This circuitry is critical for inducing adaptation of the vestibulo-ocular reflex (De Zeeuw and Yeo 2005; Lisberger et al. 1984). Unlike the vestibular nuclei and cerebellum where a wealth of literature on the computation of vestibular information has been reported (Angelaki and Cullen 2007; Angelaki et al. 2010; Chan 1997; Wulff et al. 2009), comparatively little is known about the role of the IO nuclear complex in processing vestibular information. Also, whether neurons in different IO subnuclei exhibit unique developmental profiles in processing vestibular signals remain elusive.

IO neurons are responsive to natural stimulations of the vestibular system. While otolithic inputs reach IO subnuclei dorsomedial cell column (DMCC) (Barmack 1996) and β subnucleus (IOβ) (Kaufman et al. 1991; Lai et al. 2004, 2006; Marshburn et al. 1997), the capability of IO neurons in coding linear acceleration in 3-D space remains to be elucidated. These two IO subnuclei receive vestibular-related GABAergic projections from the parasolitary nucleus (Barmack et al. 1998) and cholinergic projections from the prepositus hypoglossal nucleus (Barmack and Yakhnitsa 2000; Brown et al. 1977; Swenson and Castro 1983). Tracing studies indicated that DMCC and IOβ also receive direct projections from the medial and spinal vestibular nuclei as well as from the dorsal part of group y (Matesz et al. 2002; Saint-Cyr and Courville 1979). Nevertheless, the neurotransmitters involved in these vestibulo-olivary pathways remain unexplored. Notably, the major excitatory input to neurons in adult IO is glutamatergic in nature (Lang 2001). Such glutamatergic input to IO neurons is responsible for the expression of rhythmic neuronal activities (Du and Harvey 1997; Kitahara et al. 1995; Lang 2001, 2002; Placantonakis and Welsh 2001) that are important in modulating spatial and temporal dynamics during motor coordination (Llinás 2009). In rats, the density of ionotropic glutamate receptors in the IO region peaked at postnatal day (P) 9 and then gradually decreased to adult level by P23–30 (Rao et al. 1995). Hitherto, the expression pattern of glutamate receptors in specific IO neurons that process gravity-related movements in the 3-D space is not available. Also, the role of glutamatergic input in regulating signal transmission of vestibular-related IO neurons during postnatal development is unclear.

In rats, IO is far from mature at birth and undergoes progressive structural changes until P30 (Bourrat and Sotelo 1984; Cunningham et al. 1999). It is during P10–15 when the multiple innervations from climbing fibres to each Purkinje cell regress to the adult configuration of one climbing fibre per Purkinje cell which can then show characteristic complex spikes when triggered by IO input (Crépel et al. 1976; Mariani and Changeux 1981). In the adult, climbing fibre input from the IO is crucial for the expression of cerebellar long-term depression and vestibular-related motor learning (De Zeeuw et al. 1998). However, the spatial coding capability of IO neurons, as sole origin of climbing fibres, during the course of postnatal development remains unaddressed.

Using double immunostaining approach, we first mapped the maturation profile of IO neurons that encoded gravity-related movements in 3-D and then the expression pattern of α-amino-3-hydroxy-5-methyl-4-isoxazolepropionate (AMPA) and *N*-methyl-d-aspartate (NMDA) receptor subunits in these functionally identified neurons. NMDA receptors at the synapse are assembled as tetramers composed of two NR1 and two NR2 subunits. Given that the NR1 subunit is an obligatory component of functional NMDA receptors (Mayer and Armstrong 2004; Monyer et al. 1994), we focused on NR1 subunit in this study. The NR2 subunit, however, consists of different isoforms which are expressed differentially across cell types and developmental stages of the animal (Mayer and Armstrong 2004; Monyer et al. 1994). For AMPA receptor, the GluR2/3 subunit was examined in this study because biophysical properties of the receptor, such as permeability to calcium, are critically dependent on the subunit. This subunit also contributes to the key role of AMPA receptors in the regulation of long-term synaptic plasticity (Isaac et al. 2007). We further pursued the developmental features of glutamate receptor-mediated postsynaptic current in these neurons. Our results provide the framework for the study on efficacy of glutamatergic synapses within the otolith-related IO circuitry for developmental coding of translational head movements.

## Materials and methods

### Animal preparation

For the study of Fos expression, Sprague–Dawley rats aged P7, P9, P11, P13, P15, P17, P19, P21, P28, and adult (180–220 g) were used. Following each mode of vestibular stimulation, tissues of six rats per age group were prepared for immunohistochemical analysis of Fos protein. Tissues of another six rats per age group were prepared for double immunohistochemical analysis of Fos/NR1 co-expression or Fos/GluR2/3 co-expression. For each mode of vestibular stimulation, another set of rats aged P9, P11, P13, P21, P28, and adult (6 per age group) was used to prepare tissues for in situ hybridization analysis of target transcripts. For electrophysiological experiments, rats of P1, P4, P7, P14, and P21 (*n* = 8–14 for each group) were used. Table 1 shows the number of animals used in various experimental groups. All rats were provided by the Laboratory Animals Unit, The University of Hong Kong. In experiments using developing animals, the exact time of birth becomes critical. Pregnant females were checked daily for the presence of new litters, and the day of birth was considered to be postnatal day P0. P1 was defined as the first 24 h after birth. Prior to experiment, all rat pups were housed in a standard animal care facility and were in good health and free of ear disease. Since morphophysiological evidence indicated that vestibular nuclear neurons, the upstream relay of the vestibulo-olivary pathway, became responsive to vestibular cues around the end of the first postnatal week (Lai and Chan 2001; Lai et al. 2004, 2006; Morris et al. 1988), rats of P7–11 were studied to identify the earliest age when IO neurons could first mediate signals arising from the otolith organ. Rats of P13–17 were also studied to find if further changes occur in relation to the timeline of sensorimotor maturation from central vestibular neurons (Lai and Chan 2001) to air-righting reflex (Hård and Larsson 1975; Laouris et al. 1990). P21 was chosen for assessment since the spatiotemporal properties of neurons in the vestibular nuclei, the upstream relay of the vestibulo-olivary pathway, have attained adult levels by the third postnatal week (Lai and Chan 2001; Lai et al. 2004). Rats of P28 were also chosen because the vestibular end organs become fully mature by the end of the first postnatal month (Wubbels et al. 2002).

**Table 1:** Experimental design

	Age of animals									
	P7	P9	P11	P13	P15	P17	P19	P21	P28	Adult
Immuno-/hybridization histochemical experiments										
Normal rats										
Horizontal linear acceleration (along interaural axis)	12	18	18	18	12	12	12	18	18	18^a^
Horizontal linear acceleration (along antero-posterior axis)	12	18	18	18	12	12	12	18	18	18^a^
Vertical linear acceleration	12	18	18	18	12	12	12	18	18	18^a^
Stationary	4	4	4	4	4	4	4	4	4	4
Labyrinthectomized rats										
Horizontal linear acceleration (along interaural axis)	4	4	4	4	4	4	4	4	4	4
Horizontal linear acceleration (along antero-posterior axis)	4	4	4	4	4	4	4	4	4	4
Vertical linear acceleration	4	4	4	4	4	4	4	4	4	4
Stationary	4	4	4	4	4	4	4	4	4	4

	Age of animals				
	P1	P4	P7	P14	P21
Electrophysiological experiments					
mEPSCs	8	7	6	5	4
Subthreshold oscillations	–	7	6	5	4

Number of animals used is listed under each age group

^a^Another 16 rats for determination of optimal stimulus paradigms; another 6 rats for Western blot experiments

The immuno-/hybridization histochemistry experiments were conducted on conscious rats. All procedures conformed to the Principles of Laboratory Animal Care (NIH publication no. 86-23, revised 1985) and were approved by the University of Hong Kong Committee on the Use of Live Animals in Teaching and Research. All rats were subjected to sinusoidal linear acceleration on a moving table either along the horizontal plane or vertical plane. As described in previous studies, each conscious experimental rat was enclosed in a perspex restrainer with the head of the animal cushioned against a silicone head mask supported externally by an acrylic tube (Chen et al. 2003; Lai et al. 2004, 2010). The tube was fitted with laterally extending aluminium bars and a dorsal screw that allowed the entire head immobilization device to be positioned within the restrainer (cf. Lai et al. 1995, 2004, 2010). The trunk of each rat was also cushioned with foam padding to minimize movements during linear acceleration. Most postnatal experiments were done with littermates of the same sex and same labyrinth control (i.e. either normal or lesioned). In each experiment, one rat placed inside a restrainer was mounted onto the moving table and another placed next to the moving table was used as the static control.

### Linear acceleration paradigm

Experiments were performed in a double-walled soundproof room (NAP, Australia), which was dark and had its temperature maintained at 22 °C. To determine the optimal stimulation parameters, adult rats were subjected to sinusoidal linear acceleration along 3 perpendicular axes (antero-posterior, interaural and vertical axis) at 0.5, 1, 1.5 or 2 Hz for 30, 60, 90 or 120 min. This stimulation paradigm should effectively activate selective pairs of otolith hair cells with opposite optimal response orientations. Optimal stimulus paradigm for Fos expression refers to the stimulus parameters (frequency and duration) that resulted in plateau (or near plateau) level of Fos expression. Optimal Fos expression was observed when the animal was subjected to linear acceleration at 1.5 Hz for 90 min. These were therefore taken as the standard parameters in the present study. The rats did not show signs of struggle or stress in the course of linear acceleration. Also, they remained calm and exhibited no obvious sign of ataxia.

### Controls: ablation of bilateral labyrinths

During vestibular stimulation, increase in proprioceptive inputs and stress in rats may trigger Fos expression in their central neurons, which might be mis-interpreted as components of the vestibular circuitry. Therefore, to evaluate the possible contribution of proprioceptive inputs and stress, bilateral labyrinthectomy was performed in rats 2 weeks prior to the experiment while labyrinthectomy in postnatal stages was performed in newborn pups (1-day old) (*n* = 4 in each group). All rats were initially anaesthetized with halothane (ICI, UK) (adult 1.5–2.0 %, 250 cm^3^/min; P1 0.5–1.0 %, 100 cm^3^/min). Surgical operation was conducted with a retroauricular approach under a dissection microscope (Olympus MTX, Japan). The edge of the external ears was exposed by dissecting the thin muscle layers and the bulla was exposed with a dental drill. Subsequently, the tympanic membrane and the ear bones were removed. Special attention was paid to avoid damage to the pterygopalatine artery. The oval window was then opened and enlarged. The vestibule was aspirated with the use of a fine plastic suction pipette and then destroyed by mechanical ablation. Subsequently, the cavity of bulla and vestibule was packed with gelfoam (absorbable gelatine sponge) (Ferrosan, Sweden) and the wound was then sutured. The lesion was performed on both sides in all labyrinthectomized rats. After lesion, the operated pups were returned to their dam while the adult rats were returned to single cages kept in the same room for the normal ones. Both the normal and labyrinthectomized newborn pups were reared with their mothers until the postnatal stage for experiment. Appropriate care was provided. Both lidocaine (Astra, France) and antibiotic ointments (Furacin, South Africa) were applied 4 times daily to the skin wound. The health status of the operated animals was closely monitored. In case the operated pups showed signs of pain after lidocaine treatment, buprenorphine (Sigma, USA) was given subcutaneously. All suckling pups, including the operated ones, showed positive signs of health status, such as daily gain in body weight and stomach full of ingested mild after each meal. Rats at P5–7 showed no observable oscillatory movement of the eyeballs under their closed eyelids. There was neither nystagmus nor deviation of eye position, ascertaining the complete bilateral lesions (cf. Baloh and Halmagyi 1996). The completeness of the labyrinthine destruction was further confirmed by a post mortem examination of the temporal bone under the dissecting microscope.

Two to three weeks postoperative recovery period was allowed before the rat were subjected to linear acceleration along the horizontal plane (antero-posterior or interaural axis) or the vertical plane or kept stationary. Another group of control animal was labyrinth-intact rats that were put into the restrainers but kept stationary for 90 min before Fos immunohistochemical reactions were conducted.

### Tissue preparation

Within 5 min after linear acceleration, animals were deeply anaesthetized with Nembuital (Rhone Merieux, 60 mg/kg, ip) and perfused with normal saline via the ascending aorta. This was followed by ice-cold 4 % paraformaldehyde. Brains were postfixed for 4 h, and then cryoprotected in 20 % sucrose in phosphate buffer overnight at 4 °C. Frozen coronal sections from each rat were cut serially at 40 μm. To prevent variable results yield by different batches of materials, tissue sections used in this study were stored in cryoprotectant (0.05 M phosphate buffer, 30 % ethylene glycol, 20 % glycerol; at −20 °C) such that sections of different sections of different age groups were processed at the same time. To demarcate the boundaries of cell groups of interest, one-in-two series of brainstem sections throughout the IO were collected. These were processed alternately for Nissl staining and Fos immunohistochemistry. Those sections for Nissl staining were mounted on slides, air-dried, and placed in Nissl stain for 5–10 min. The tissues on the slides are then dehydrated and cover slipped with DPX (BDH, UK) as a mounting medium. For experiments investigating the co-expression of Fos with glutamate receptor subunit (NR1 or GluR2/3), one-in-two series of brainstem sections were collected. These were processed alternately for double immunostaining, either for Fos/NR1 or for Fos/GluR2/3. One-in-three series of brainstem sections were used for in situ hybridization analysis of NR1, NR2A and NR2B mRNAs.

### Primary antibodies

Commercial antibody against *c*-*fos* (1:600; goat polyclonal against a synthetic *N*-terminal fragment of human Fos; sc-52G; Santa Cruz Biotechnology, USA), NR1 (0.5–1.0 μg/ml; rabbit polyclonal that was specific to four spice variants of NR1 receptors; AB1516; Chemicon, USA) or GluR2/3 (1 μg/ml; rabbit polyclonal; AB1506; Chemicon, USA) were used for both immunohistochemistry and Western blotting. Specificity of staining was verified by either replacing the primary antibody with normal serum or preabsorbing the antibody with its corresponding immunizing peptide (sc-52P for *c*-*fos*, Santa Cruz Biotechnology, at 1 μg peptide per 1 μl anti-serum; AG344 for NR1; AG305 for GluR2/3, Chemicon, USA, at 1 μg peptide per 5 μl anti-serum).

Neural tissues from the IO complex and two control regions, viz. dorsal medulla and cerebral cortex, were collected. These freshly dissected issues from each brain region pooled from 6 adult rats were separately homogenized in lysis buffer. The homogenate was centrifuged at 14,000*g* at 4 °C for 1 h. The supernatant was subjected to electrophoresis in 8 % sodium dodecylsufate-polyacrylamide gel and then electroblotted onto nitrocellulose membrane. Blots were first incubated with 5 % nonfat powdered milk dissolved in Tris-buffered saline for 1 h to block nonspecific binding sites and then probed with either polyclonal or monoclonal antibody for 2 h. Each blot was treated with either a horseradish peroxidase-linked secondary antibody against rabbit IgG (1:1,000 in Tris-buffered saline; Zymed Laboratories, USA), horseradish peroxidase-linked secondary antibody against goat IgG (1:1,000 in Tris-buffered saline; Zymed Laboratories, USA) or horseradish peroxidase-linked secondary antibody against mouse IgG (1:1,000 in Tris-buffered saline; Zymed Laboratories) for 1 h. Finally, bands were visualized with the ECL-Western blotting analysis system (Amersham, USA). Incubation with the *c*-*fos* antibody revealed a single immunoreactive band of ~57 kDa protein. NR1 and GluR2/3 receptor subunits revealed single bands of ~120 and ~108 kDa, respectively. These bands were not revealed when blots were probed with antibody that had been neutralized by preincubation with the respective immunizing peptide or in those blots that were incubated in Tris-buffered saline in place of the primary antibodies.

For NR1 subunit, the specificity of immunostaining in IO neurons was further confirmed by combining with in situ hybridization for the respective mRNA. Also, for NR2A and NR2B subunits, in which the corresponding immunizing peptide was not available, combined immuno- and hybridization histochemistry was performed to verify the specificity. In brief, cRNA probes for NR1, NR2A and NR2B were generated from cDNAs encoding NR1 (4.2 kb), NR2A (4.2 kb) and NR2B (4.5 kb). These cDNAs, provided by Dr. R.K.W. Chan (Laboratory of Neuronal Structure and Function, The Salk Institute, La Jolla, CA, USA), corresponded in sequence to those reported in GenBank (NR1: accession No. X63255; NR2A: accession No. M91561; NR2B: accession No. M91562). Plasmids containing the cDNA clone were linearized with *Hin*dIII (for NR2A; Promega Corporation, Madison, WI, USA) or *Sph*I (for NR1 and NR2B; Promega Corporation). Antisense cRNA probes labeled with [α-^35^S]UTP (NEN, USA) were synthesized using T3 (for NR1 and NR2A) and T7 (for NR2B) RNA polymerase (Promega Corporation, USA) following linearization of the plasmids (pBluescript SK-) with restriction enzymes. Unincorporated nucleotides were removed by Quick Spin columns (Boehringer Mannheim, USA). The specific activities of the [α-^35^S]UTP probes were on the order of 2 × 10^9^ dpm/μg. All restriction enzymes and RNA polymerases were obtained from Promega Corporation. Our result confirmed that staining with each of three antibodies was co-localized with in situ hybridization for the respective mRNA.

### Immunohistochemistry

Fos immunohistochemistry was conducted on free-floating coronal serial brainstem sections (Chen et al. 2003). Frozen coronal brainstem sections collected consecutively from each animal were divided in series in which every alternative section was treated alike. Each series was immunostained for Fos and in turn for NR1 or GluR2/3. After being rinsed in potassium phosphate buffer saline (KPBS) to remove cryoprotectant, nonspecific binding sites of floating sections were blocked in normal donkey serum (NDS 1:50) for 30 min at room temperature. Then, primary Fos antibody (1:600, sc-52G Santa Cruz Biotechnology, USA) with 2 % normal goat serum and 0.3 % Triton X-100 in KPBS was applied overnight at room temperature. This was followed by secondary antibody (1:400, biotinylated goat anti-rabbit IgG; Vector, USA) for 2 h, and avidin–biotin peroxidase complex (1:100; Vector, USA) in PBS for 1 h at room temperature. Between each step, the sections were rinsed three times with KPBS (5 min/rinse). During each step, the sections were agitated on a rotator. Finally, an intensified diaminobenzidine (DAB) reaction (DAB, 0.02 % w/v; H_2_O_2_, 0.002 % v/v; in KPBS; Vector, USA) was carried out for 5–10 min at room temperature.

Double immunohistochemistry was performed immediately after the Fos labeling intensified with DAB. The sections were incubated in primary antibody solutions against different glutamate receptor subunits with 2 % normal goat serum and 0.3 % Triton X-100 in PBS for overnight at room temperature. Then this was followed by secondary antibody (biotinylated IgG, 1:400, Vector, USA) for 2 h, and avidin–biotin peroxidase complex (Vector, USA) in PBS for 1 h at room temperature. In-between each step, the sections were rinsed three times with PBS (5 min/rinse) and then agitated on a rotator. Finally, the sections were intensified with SG Substrate Kit (Vector, USA) in PBS for 5–10 min at room temperature. The consecutive brainstem coronal sections were then mounted on gelatin-coated slides, dried, dehydrated and coverslipped.

Control and experimental tissues from each group were processed in parallel. No staining was observed on brainstem sections with the omission of either the primary or the secondary antibody. Different age groups of brainstem slices were also processed at the same time.

### Cell counting and statistical analysis

The number of Fos-immunoreactive (Fos-ir) nuclear profile within the confines of each of the anatomically demarcated cell groups in all animals was evaluated with bright-field microscope in complete series of coronal sections through cell groups of interest. In each group of rats, only cells on both sides that exhibited significant levels of DAB reaction product in their nucleus greater than that of tissue background levels were counted. The number of Fos-ir cells in each region was estimated using a digital image analysis system (version 1.61, W. Rasband, NIH, USA). Tissue background signals were averaged over defined areas of cell aggregrate in Fos-ir-poor areas. Average tissue background was set at the minimum on a gray scale of 255 shades. Threshold was then established at the gray level of 190 units (cf. Kaufman et al. 1993; Janusonis and Fite 2001; Lai et al. 2006, 2010; Moratalla et al. 1996; Tse et al. 2008). In each experimental and control animal, positive Fos labelling was confirmed when the darkly stained nuclei of Fos-ir neurons reached a gray level above the threshold.

For each animal, the number of cell counted in each brain area of interest was divided by the number of tissue sections containing that area to give a mean cell count per animal per area. In addition, estimates were corrected for double-counting errors using the method of Abercrombie (1946). The classification of anatomically demarcated cell groups conformed to the description in the brain atlas of neonatal (Paxinos et al. 1994) and adult rats (Paxinos and Watson 1998). The relative location and morphology of brainstem nuclei of postnatal rats were very similar to those of the adult. In DMCC, cell number was counted from both sides since the boundary between the left and right side at the rostral end of the DMCC could not be clearly distinguished. The cell number in the DMCC was presented in a unilateral manner, by halving the counted number. In the other regions, cell number was counted on either side.

Fos-ir neurons and their mRNA signal images were captured by a microscope (Axioplan II, Carl Zeiss, Germany) with a CCD camera (Spot, Diagnostic Instrument, USA) under bright field and dark field, respectively, at high magnification. These signals were analyzed by an imaging analyzing system (MetaMorph Ver 4.01, Universal Imaging Corp., USA). In each tissue section, only granule density (the number of granules over the counted area) exceeding three times background level was defined as positive hybridization signal. To determine whether granule density values lay within the linear range of the detection system, brain paste standards were prepared.

Data presented are mean ± SEM. Student’s *t* test was used to compare differences in the mean number of Fos-ir nuclei/cell group between normal and labyrinthectomy control. The mean number of Fos-ir nuclei/cell group at each specific stage of postnatal development was compared using one-way ANOVA followed by Tukey–Kramer multiple comparisons test. In all analyses, a probability value of *P* < 0.05 was taken to be statistically significant.

### In vitro electrophysiological studies

Rats are decapitated under isoflurane anaesthesia. The brainstems were immediately removed to ice-cold ACSF composed of (in mM) 120 NaCl, 2 KCl, 2.5 CaCl_2_, 1.2 MgCl_2_, 1.2 KH_2_PO_4_, 26 NaHCO_3_ and 11 glucose, saturated with 95 % O_2_ and 5 % CO_2_, pH 7.4. Coronal brainstem slices (300 μm) that contain the IO were prepared using a vibrotome (MA752, Campden Instruments, UK). Neurons perfused with above ACSF were monitored through a CCD camera on a microscope equipped with infrared-differential interference contrast optics. Whole-cell recordings were made with patch pipettes (3–6 MΩ) filled with (in mM) 130 Cs-gluconate, 2 MgCl_2_, 2 NaCl, 5 K_2_ATP, 1 EGTA, 10 HEPES from visually identified neurons using a Multiclamp-700A amplifier (Axon Instruments, USA). Change of series resistance is <15 %. Data are captured by Digidata-pClamp package. Automatic detection and analysis of miniature EPSCs were performed using MiniAnalysis program 6.0.3 (Synaptosoft, USA). In recording miniature EPSCs, Mg^2+^-free perfusate was used while action potential, GABA_A_ receptor-/glycine receptor-mediated currents were blocked by adding 1 μM TTX, 10 μM bicuculline and 1 μM strychnine, respectively. EPSCs were recorded at a holding potential of −70 mV. Selective receptor antagonists, AP5 for NMDA receptors and CNQX for AMPA receptors were used to identify the glutamate receptor involved in the expression of miniature EPSCs. Subthreshold oscillation in membrane potential of IO neuron was recorded under current-clamp mode. Micropipette was filled with: (in mM) 130 Cs-gluconate, 2 MgCl_2_, 2 NaCl, 5 K_2_ATP, 1 EGTA, 10 HEPES. Only neurons with a membrane potential negative to −50 mV were analyzed. The results were processed off-line with Clampfit 9.0 (Axon Instruments, USA). All data were computed and presented as mean ± SEM. The unpaired *t* test or ANOVA followed by post hoc Scheffe’s test was used for statistical analysis as appropriate.

## Results

### Fos expression: stimulus parameters and control experiments

**Fig. 1:**
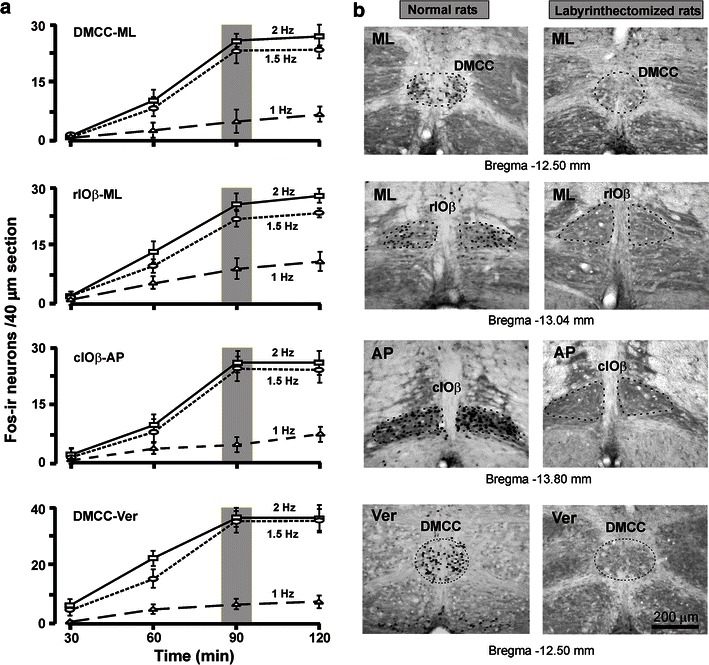
**a** Stimulus paradigm for Fos expression within DMCC and IOβ in normal adult rats that were subjected to sinusoidal linear acceleration on the horizontal plane (along antero-posterior or interaural axis) or vertical plane at different frequencies (1, 1.5 or 2 Hz) and for different durations (30, 60, 90 or 120 min). The optimal paradigm chosen for the present study was 1.5 Hz for 90 min (*shaded area*). **b** Photomicrographs showing the distribution of Fos-ir neurons in IO subnuclei of normal (*left column*) and labyrinthectomized (*right column*) rats. These animals were subjected to sinusoidal horizontal linear acceleration along interaural axis (*ML*) or antero-posterior axis (*AP*). The value under each panel represents the distance of the section caudal to the Bregma. Note that no Fos-ir neurons were observed in the DMCC and IOβ of labyrinthectomized rats. *DMCC* dorsomedial cell column, *cIOβ* caudal part of beta nucleus, *rIOβ* rostral part of beta nucleus, *AP* antero-posterior stimulation, *ML* interaural stimulation, *Ver* vertical stimulation

Fos protein has been used as a neural marker to identify functionally activated otolith-related IO neurons in rats following sinusoidal linear acceleration along the horizontal or vertical plane. In histological sections, Fos immunostaining was exclusively in cell nuclei which were seen as dark, round or oval structures (Fig. 1). There was no significant difference in the number of Fos-ir neurons between littermates or between sexes of the same age group of experimental rats. In vast majority of rats, the Fos expression pattern was symmetrical on either side of the brainstem after either mode of otolith stimulation. In a few odd cases, asymmetric cell count on the two sides was observed but the asymmetry was not restricted to any side and thus not related to the specific direction of stimulation. These cases were not included for analysis.

To determine the optimal stimulus paradigm for Fos expression in the IO, experiments were performed on adult animals (*n* = 4 for each mode of stimulation) subjected to linear acceleration (at 0.5, 1.0, 1.5, or 2.0 Hz) along the horizontal or vertical plane (for 30, 60, 90, or 120 min). Figure 1 shows the expression pattern of Fos induced by sinusoidal linear acceleration along the interaural axis on the horizontal plane. With increase in frequency or duration of stimulation, progressive increase in the number of Fos-ir neurons was observed in IO subnuclei. No Fos expression was observed in rats stimulated at 0.5 Hz. Optimal Fos expression emerged at 1.5 Hz stimulation for 90 min (Fig. 1). Thus, these were chosen as the standard parameters for Fos expression in our study.

In the present study, we confirmed that the expression of Fos protein was due to the activation of hair cells on the utricular or saccular maculae by performing control experiments in which normal rats were kept stationary (*n* = 4). This is corroborated by the absence of Fos expression in labyrinthectomized rats that were subjected to different modes of linear acceleration (*n* = 4 in each mode of stimulation) and in labyrinthectomized rats that remained stationary (*n* = 4). In labyrinthectomized rats subjected to different modes of linear acceleration, Fos-ir neurons were either absent or only sporadically found (<3 cells/nucleus/section) in IO subnuclei, DMCC and IOβ (Fig. 1). The same results were observed in stationary subgroups of normal and labyrinthectomized rats. In normal experimental rats, Fos expression was only taken as an indicator of otoltihic input when the number of Fos-ir neurons in any one serial section of these IO subnuclei was ≥4.

In subnuclei IOB and IOC, however, a significant number of Fos-ir neurons was observed even in labyrinthectomized rats. Therefore, Fos expression in IOB and IOC subnuclei was not considered as triggered by the otolith end organ. It is noteworthy that in stress-related brainstem nuclei (Chen and Herbert 1995; Cullinan et al. 1995), such as the locus coeruleus, moderate number of Fos-ir neurons (on average 3–8 cells/nucleus/section) was observed even in control preparations. In control rats (P7, adult) in which neurons in the vestibular nuclei exhibited no Fos labeling, the periaqueductal gray had 20–25 Fos-ir cell/section, indicating that the Fos expression so observed was not due to vestibular input. When the primary antibody was omitted from the protocol, Fos labeling was not observed in these areas. Fos expression in these areas was thus taken as an internal indicator of working Fos immunocytochemistry in the brainstem.

### Distribution pattern of Fos-ir IO neurons in adult rats

**Fig. 2:**
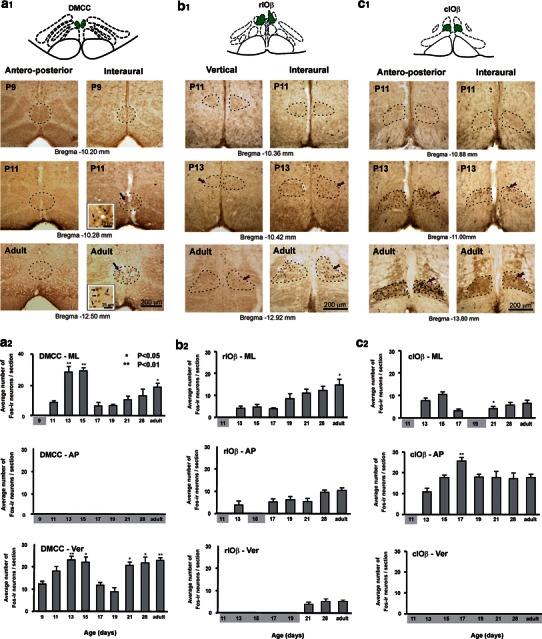
Distribution and number of For-ir neurons in DMCC (**a**), rIOβ (**b**) and cIOβ (**c**) of postnatal and adult rats subjected to horizontal linear acceleration (along the antero-posterior or interaural axis) or vertical linear acceleration. **a**_**1**_–**c**_**1**_ Photomicrographs showing the distribution of Fos-ir neurons in 3 age groups. No Fos-ir DMCC neuron was observed in rats subjected to antero-posterior stimulation in all age groups. Note also the absence of Fos-ir neuron in rIOβ and cIOβ at P11. *Circled areas* are shown at higher magnification in *insets*. *Arrows* indicate examples of Fos-ir neurons. **a**_**2**_–**c**_**2**_ The number of Fos-ir neurons (per 40 μm section) in IO subnuclei of different age groups of rats that were subjected to the respective linear accelerations at 1.5 Hz for 90 min. In DMCC, For-ir neurons activated by interaural or vertical linear acceleration showed a significant decrease in number at P17 and then exhibited a progressive increase during the 4th postnatal week. Similar observation was found in cIOβ neurons activated by interaural linear acceleration. *Asterisks* indicate the probability levels for significant difference between age groups: rIOβ and cIOβ compared with P13 rats; DMCC compared with P9 for vertical stimulation and with P11 for ML stimulation. Age groups in which IO subnuclei exhibited no significant Fos labeling were highlighted. *DMCC* dorsomedial cell column, *cIOβ* caudal part of beta nucleus, *rIOβ* rostral part of beta nucleus, *AP* antero-posterior horizontal stimulation, *ML* interaural horizontal stimulation, *Ver* vertical stimulation

**Fig. 3:**
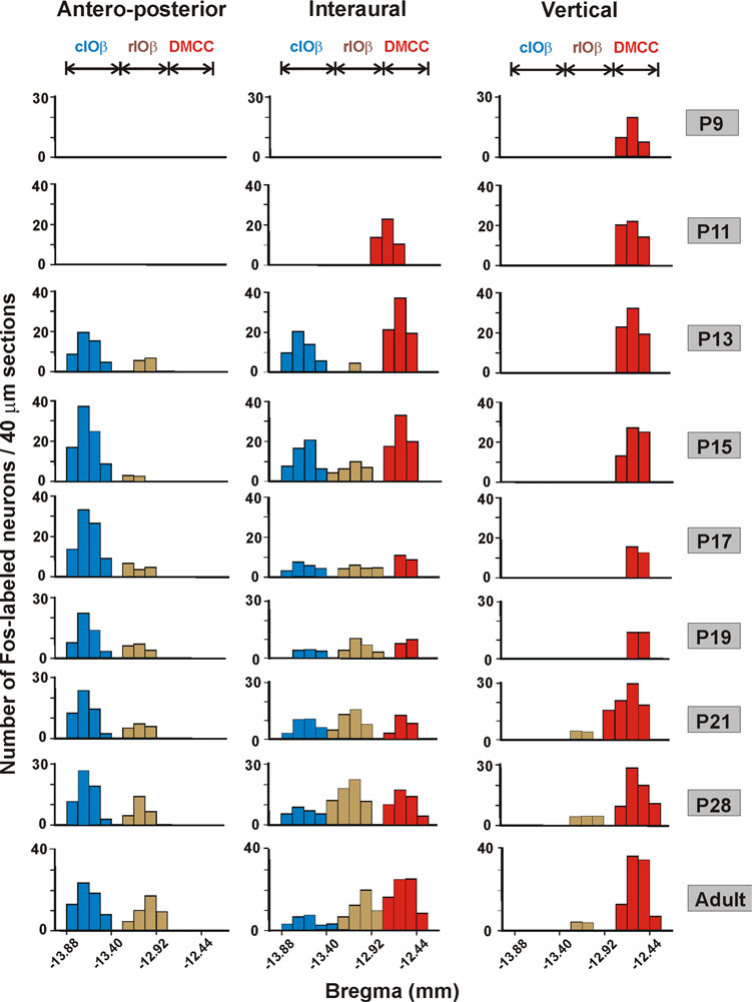
The number of Fos-ir neurons in DMCC and IOβ of different age groups of rats subjected to horizontal linear acceleration (along the antero-posterior or interaural axis) or vertical linear acceleration at 1.5 Hz for 90 min. *Each column* represents the average number of Fos-ir neurons at defined levels of the brainstem. *DMCC* dorsomedial cell column, *rIOβ* rostral part of beta nucleus, *cIOβ* caudal part of beta nucleus

Fos protein expression in each IO subnucleus was examined in adult rats (*n* = 6 in each mode of linear acceleration) that were subjected to sinusoidal linear acceleration along the horizontal (antero-posterior or interaural) or vertical direction. No Fos expression was observed in subnuclei IOA, IOK, IOD, IOPr, IOM, and IOV while Fos expression in IOB and IOC subnuclei was discounted as argued above. Within DMCC and IOβ, different patterns of Fos expression corresponding to spatial orientations in 3-D were observed (Fig. 2). In the DMCC (12.32–12.8 mm caudal to Bregma), a large number of Fos-ir neurons was observed in the middle portion while a relatively small number was found in either ends. Similar distribution was found within IOβ (12.8–13.88 mm caudal to Bregma) (Fig. 3).

Horizontal linear acceleration along the interaural axis activated neurons in DMCC, rostral IOβ (rIOβ) and caudal IOβ (cIOβ). A significantly smaller number of Fos-ir neurons was found in the cIOβ. With linear acceleration along the naso-occipital axis, Fos-ir neurons were only found in the rIOβ and cIOβ. The number of these neurons was lower in the rIOβ (Fig. 2). With linear acceleration along the vertical axis, Fos-ir neurons were mainly found in DMCC and a relatively small number in rIOβ. It is noteworthy that the number of Fos-ir DMCC neurons activated by vertical linear acceleration was significantly higher than that by horizontal interaural linear acceleration (Fig. 2). Taken together, it is evident that DMCC encodes vertical orientations as well as horizontal orientations along the interaural axis. Caudal IOβ, on the other hand, only encodes horizontal stimulations. Coding in 3-D is restricted to neurons in the middle region of rostral IOβ (Fig. 3).

### Fos expression in developmental rats subjected to horizontal and vertical linear acceleration

P9 was the earliest age by which gravity-related spatial orientation was encoded in the IO (Fig. 2a). At this age, For-ir neurons activated by vertical linear acceleration were identified in the DMCC (11 ± 1 cells/section). The number of these neurons increased until P13–15 (23 ± 1 cells/section). Neurons in DMCC became also responsive to horizontal (interaural) stimulation at P11 (8 ± 2 cells/section). The number of these DMCC neurons also increased with age and peaked between P13 and P15 (28 ± 2 cells/section). Interestingly, Fos-ir DMCC neurons activated by these modes of stimulation exhibited a significant decrease in number at P17 (vertical 9 ± 1 cells/section; interaural 6 ± 2 cells/section) and kept at a low level at P19 (vertical 10 ± 1 cells/section; interaural 7 ± 1 cells/section). Nevertheless, progressive increase was evidenced after P21 (vertical 18 ± 2 cells/section; interaural 10 ± 2 cells/section) until adulthood (vertical 28 ± 2 cells/section; interaural 18 ± 3 cells/section) (Fig. 2a_2_). Notably, DMCC neurons were not responsive to horizontal antero-posterior stimulation in all age groups.

In IOβ, Fos-ir neurons could only be identified from P13 onwards, i.e. 2 days later than in the DMCC (Fig. 2b, c). rIOβ neurons that were responsive to horizontal interaural (5 ± 1 cells/section at P15) and antero-posterior stimulation (4 ± 1 cells/section at P13) increased with age, reaching adult pattern at P28 (interaural 15 ± 3 cells/section; anterior-posterior 10 ± 1 cells/section) (Figs. 2b_2_, 3). For-ir neurons responsive to vertical linear acceleration were also found in the middle part of rIOβ from P21 onwards though the cell number was relatively small (Fig. 3). Altogether, these findings indicate that coding of spatial orientations in 3-D was evidenced within rIOβ from P21 onwards.

In the cIOβ, neurons responsive to horizontal antero-posterior stimulation (10 ± 2 cells/section in P13) increased with age, peaked at P17 (25 ± 2 cells/section), and dropped to a stable level from P19 (18 ± 1 cells/section) onwards (Figs. 2c_2_. 3). On the other hand, cIOβ neurons responsive to horizontal interaural stimulation displayed a maturation profile comparable to that of DMCC neurons responsive to the same stimulation (Figs. 2a_2_, 3). The number of Fos-ir neurons in cIOβ dropped significantly from P13 (8 ± 1 cells/section) to P17/P21 (4 ± 1 cells/section) and maintained at a low level in adult (6 ± 1 cells/section) (Figs. 2c_2_, 3).

### Expression of glutamate receptor subunits in Fos-ir IO neurons of postnatal rats

**Fig. 4:**
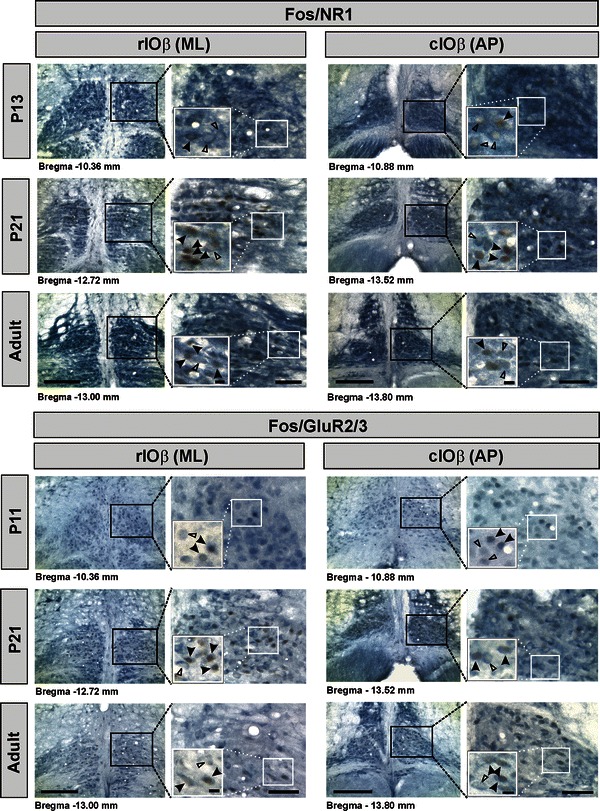
Photomicrographs showing the co-expression of Fos and glutamate receptor subunits in IOβ neurons of postnatal and adult rats subjected to horizontal linear acceleration along interaural axis (ML) or antero-posterior axis (AP). Fos-ir cell nuclei are stained *dark brown*, while immunoreactivity of glutamate receptor subunits is revealed by *dark blue*. *Filled triangles* indicate double-labeled neurons. *Open triangles* indicate neurons singly labeled with glutamate receptor. *Boxed areas* (*in black*) *on the left* are shown at higher magnification *on the right* for each pair. *Boxed areas* (*in white*) are shown at higher magnification in *insets*. The *value under each pair* represents the distance of the section caudal to the Bregma. *cIOβ* caudal part of beta nucleus, *rIOβ* rostral part of beta nucleus. *Scale bar* 200 μm (*left*) and 50 μm (*right*) for each pair; 10 μm in *insets*

Unlike Fos immunoreactivity in cell nuclei, immunoreactivity for NMDA or AMPA receptor subunits was predominantly expressed in neuronal somata and its proximal dendrites. Double immunolabeling data revealed that Fos/NR1 and Fos/GluR2/3 double-labeled neurons in the DMCC and IOβ constituted 100 % of the total Fos-ir neurons in P11 and P13 rats (Fig. 4). With increase in age, the percentage of Fos/NR1 double-labeled neurons hovered around this range. With increase in age, the percentage of double-labeled neurons in DMCC and IOβ ranged between 98 and 100 %.

**Fig. 5:**
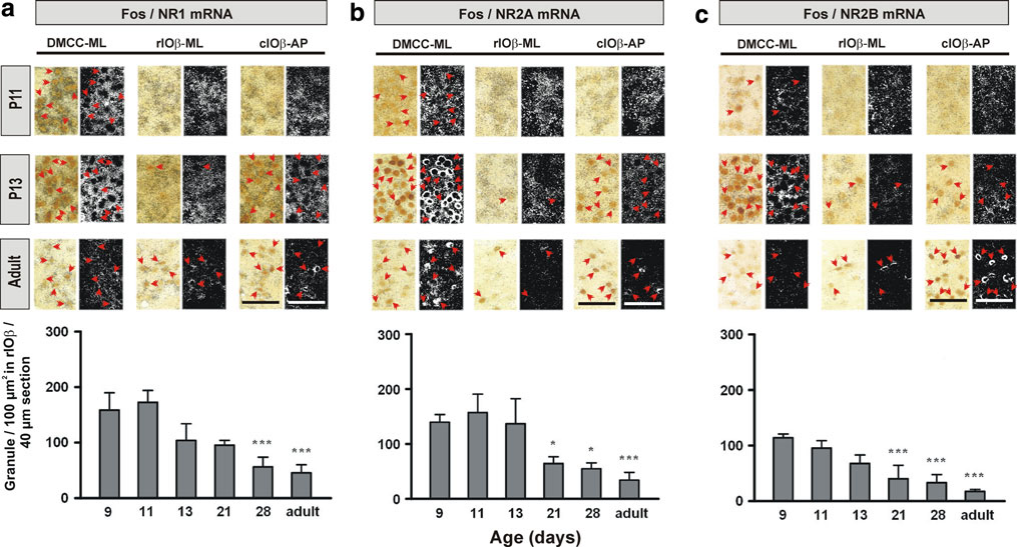
The expression pattern of NR1 mRNA (**a**), NR2A mRNA (**b**) and NR2B mRNA (**c**) on Fos-ir neurons in postnatal and adult rats subjected to horizontal linear acceleration along the interaural axis (ML) or the antero-posterior axis (AP). In each pair of photomicrographs in the *top panel*, *picture in the left* is bright-field image for Fos-ir neurons and that *in the right* is dark field image for the respective mRNA granules. *Arrowheads* indicate double-labeled neurons. In DMCC, Fos-ir neurons were first observed at P11. In IOβ, Fos-ir neurons were first observed at P13. The *lower panel* shows the granule density for the respective mRNAs in rostral IOβ of postnatal and adult rats. *Asterisks* indicate significant difference (*P < 0.05; ***P < 0.001) when compared with P9 rats. *DMCC* dorsomedial cell column, *cIOβ* caudal part of beta nucleus, *rIOβ* rostral part of beta nucleus. *Scale bar* 50 μm

Co-expression of NMDA receptor subunit mRNAs with Fos-ir neurons in DMCC and IOβ was also measured (Fig. 5). In all age groups studied, 96–99 % of Fos-ir neurons in DMCC and IOβ were defined as NR1 mRNA positive, 94–100 % as NR2A mRNA positive, and 95–100 % as NR2B mRNA positive. In DMCC, the granule density of NR1 mRNA progressively increased, peaked at P11, and then decreased as the animal matured. For NR2A mRNA, a relatively high granule density was observed during the second postnatal week, and then progressively decreased with age. Compared with NR1 and NR2A mRNAs, a relatively low granule density of NR2B mRNA was observed in DMCC and it progressively decreased with maturation. In IOβ, the developmental expression pattern of NMDA receptor subunit mRNAs was similar to that in DMCC. No significant difference was observed between rIOβ and cIOβ. The granule density of NR1 mRNA was high at P9 and P11 and then decreased with age (Fig. 5a). The granule density of NR2B mRNA decreased during postnatal development (Fig. 5c). For NR2A mRNA, the granule density in rIOβ was high at P9–13 but significantly decreased at P21 (Fig. 5b). The density in cIOβ, however, was low at P9, peaked at P11–13 and significantly decreased at P21.

### Postnatal properties of IO neurons receiving glutamatergic inputs

**Fig. 6:**
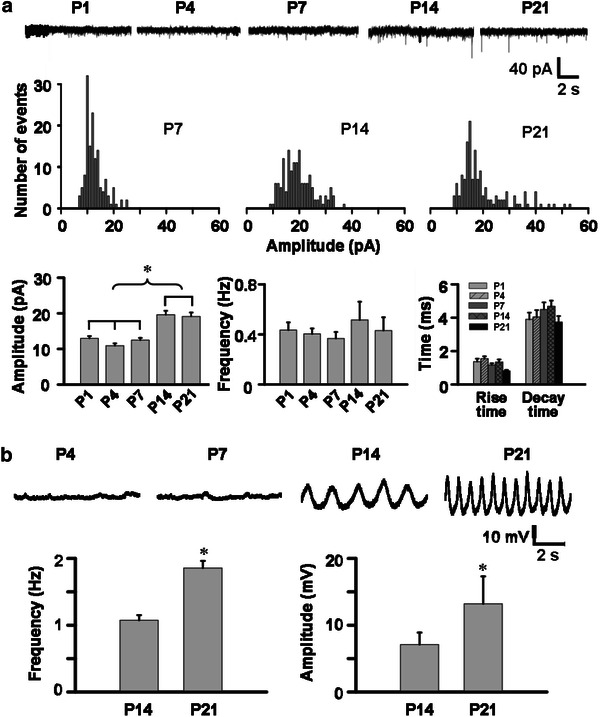
Development profile of mEPSCs (**a**) and subthreshold oscillations (**b**) of neurons in the rostral IOβ. **a** *Upper row* representative recordings from postnatal rats of indicated ages. *Middle row* representative histograms of mEPSC amplitude (bin width 1 pA; events extracted from 5-min recordings). *Bottom row* histograms showing postnatal increase in the mean amplitude of mEPSCs (*n* = 8 cells in each age group; *P* < 0.05) (*left panel*). No change was observed for the frequency, rise time and decay time of mEPSCs during postnatal development (*middle and right panels*). **b** Subthreshold oscillation of membrane potential was found as from P14. Significant increases in both the frequency and amplitude of the oscillations from P14 to P21 were observed (*n* = 8 cells in each age group; *P* < 0.05)

Whole-cell patch-clamp data were obtained from rostral IOβ neurons in brainstem slices of P1, P4, P7, P14, and P21 rats. mEPSCs recorded could be blocked with the application of 10 μM CNQX (AMPA receptor antagonist) to the bathing medium. This finding suggests that the mEPSCs of neurons in the rostral IOβ are mediated by AMPA receptors. The amplitude of mEPSC in P14 rats (19.6 ± 1.1 pA) was significantly greater than those of other age groups (13.0 ± 0.7, 10.9 ± 0.6, 12.5 ± 0.6 pA for P1, P4, P7, respectively; *P* < 0.05) (Fig. 6a). Amplitude histogram also demonstrated the increase in amplitude of mEPSCs from P7 to P21, suggesting enhancement in the size of quantal release during this stage. The amplitude of mEPSCs in P21 rats (19.1 ± 1.1 pA), however, showed no significant difference with that of P14 rats. The decay time increased while the rise time decreased with maturation. However, there was no change in the frequency of glutamate-mediated mEPSC during development. Rostral IOβ neurons showed oscillation of membrane potential from P14 onwards. Both amplitude and frequency of these oscillations at P21 were significantly greater than those of P14 rats (amplitude: 13.2 ± 4.1 mV of P21 vs. 7.1 ± 1.8 mV of P14, *P* < 0.05; frequency: 1.9 ± 0.1 Hz of P21 vs. 1.1 ± 0.1 Hz of P14, *P* < 0.05) (Fig. 6b). Such oscillation of membrane potential was not observed in rostral IOβ neurons of younger rats.

## Discussion

**Fig. 7:**
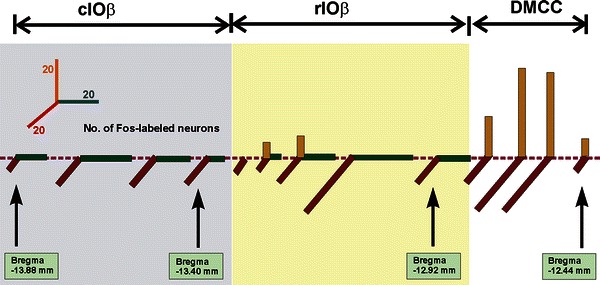
3-D histograms showing the number of Fos-ir neurons, at regular intervals throughout the rostro-caudal dimension of DMCC and IOβ in adult rats, responsive to linear accelerations along the horizontal interaural axis (ML, *red columns*), horizontal antero-posterior axis (AP, *green columns*) or vertical axis (*orange columns*). *DMCC* dorsomedial cell column, *cIOβ* caudal part of beta nucleus, *rIOβ* rostral part of beta nucleus

The present results demonstrate the existence of gravity-related 3-D topography of spatial orientations in IO subnuclei DMCC and IOβ (Fig. 7). We also documented a cascade of maturation time in which different neuronal subpopulations of IO began to encode head orientations in 3-D. Furthermore, we documented postnatal increase in the expression of AMPA and NMDA receptor subunits in otolith-related IO neurons. This is corroborated by postnatal increase in the amplitude of glutamate receptor-mediated postsynaptic current in these neurons, implying that developmental fine tuning of glutamatergic transmission along the vestibulo-olivary circuit enables coding of 3-D orientations.

### Distribution pattern of Fos-ir neurons in adult rats

Topographic organization is a ubiquitous phenomenon in sensory systems (Kaas 1997; Weinberg 1997). In the central vestibular system, canal- and otolith-inputs are in fact topographically segregated in IO subnuclei. IOβ receives only otolith input (Lai et al. 2004) while IOA, IOC and IOK receive exclusively canal input (Lai et al. 2010). DMCC is the only IO subnucleus that receives inputs from both canal (Lai et al. 2010) and otolith organ (Lai et al. 2004). In the present study, we further provided evidence that gravity-related spatial information is represented in a topographic fashion within subnuclei DMCC and IOβ. Neurons within the rostral IOβ are capable of encoding gravity-related spatial information in 3-D. Based on the functional polarization vectors of hair cells on the utricular and saccular macula (Fernández and Goldberg 1976), our results indicate that inputs arising from the anterio-posterior and medio-lateral sectors of the utricle and the dorso-ventral portions of the saccule reach the rostral IOβ. Whether or not inputs from all three axes converge onto single neurons in the rostral IOβ awaits further electrophysiological experiments for confirmation. On the other hand, neurons in the DMCC and caudal IOβ can only encode spatial information in 2-D. Those in the caudal IOβ should receive inputs from all sectors of the utricle as well as from saccular regions that bend horizontally (Curthoys et al. 1999). Those in the DMCC, however, should receive inputs from the medio-lateral sectors of the utricle as well as from the saccule. The latter conforms to the finding that hypergravity stimulus that activated the saccule produced Fos immunolabeling in the DMCC of gerbils and rats (Gustave Dit Duflo et al. 2000; Marshburn et al. 1997).

This topographic spatial reference in the IO is very likely to be retained in the cerebellum. In view of the modular topography of olivo-cerebellar projection from individual IO subnuclei to specific strips of the cerebellar cortex (Bernard 1987), otolith signals carried by discrete IO ascending pathways should reach specific modules of Purkinje cells as “private lines”, regulating the timing and learning operation within the olivo-cerebellar system (de Zeeuw et al. 1998). Climbing fibres arising from DMCC are known to terminate in a lateral zone of uvula (Apps 1990) while those from IOβ terminate within the medial sagittal zone A of nodulus, extending into the uvula (Apps 1990; Groenewegen and Voogd 1977). Purkinje cells in these two zones in turn project to the lateral vestibular nucleus (Fushiki and Barmack 1997; Groenewegen et al. 1979) and medial vestibular nucleus, respectively (Voogd et al. 1996). We further reason that this olivo-cerebello-vestibular nucleus feedback circuitry is also topographic in nature, such that translational signals of specific directions in the 3-D plane after processing in the olivo-cerebellar pathway are fed back to specific parts of the vestibular subnuclei that encode comparable gravity-related translational signals.

Within the medial vestibular nucleus, neurons activated by linear acceleration along the *x*, *y* and *z* axes indeed showed topographic distribution (Lai et al. 2006; Zhang et al. 2005). In adult rats, medial vestibular neurons activated by vertical linear acceleration were confined to a strip spanning from the medial to lateral end of the subnucleus (Fig. 4 of Lai et al. 2006) while those activated by horizontal (medio-lateral) linear acceleration were mainly located ventral to the above strip and clustered in the ventro-lateral part of this subnucleus bordering the spinal vestibular nucleus (Fig. 2 of Zhang et al. 2005). In the spinal vestibular nucleus, neurons in the rostral end (11.1–11.4 mm caudal to Bregma; ventral to the lateral vestibular nucleus) were exclusively responsive to vertical linear acceleration but those responsive to linear acceleration along the three axes were intermingled throughout the mid-/cadual part of the subnucleus. It is anticipated that the topographic olivo-cerebellar feedback to the vestibular nucleus when matched with the corresponding topography at the vestibular nucleus level could promote smooth execution of vestibular-related sensorimotor commands.

### Maturation profile of Fos-ir neurons in postnatal rats

During postnatal development, neurons in IO can first encode gravity-related spatial information along the vertical plane at P9 but take two more days before they can encode spatial information on the horizontal plane (Fig. 2). Together with our previous data on the maturation profile of otolith-related vestibular nuclear neurons (Lai et al. 2004, 2006, 2008; Tse et al. 2008), we have revealed along the otolith-vestibulo-olivary pathway a cascade of maturation time in the recognition of gravity-related spatial orientation in 3-D. These temporal profiles, however, contrast with those within the canal-vestibulo-olivary pathway. First, Fos-ir IO neurons activated by horizontal rotations were identifiable by P4 (Lai et al. 2010) but those by vertical linear acceleration were only at P9, indicating that IO neurons acquire their capacity to encode rotational movement well before linear motion.

In developing rats, a substantial decrease in IO cell number has been reported at P14–15 (Cunningham et al. 1999). This has been attributed to neuronal death at the time when there is a loss of multiple climbing fibre innervation to each Purkinje cell (Crépel et al. 1976; Mariani and Changeux 1981). In the present study, transient decrease of Fos-ir neurons in DMCC was observed between P14 and P17 (Fig. 2). Whether such a remarkable decrease in otolith-related DMCC neuron number around P17 is also associated with the establishment of one-to-one olivo-cerebellar connection between climbing fibre and its target Purkinje cell remains to be confirmed.

### Postnatal expression of ionotropic glutamate receptors in otolith-related IO neurons

In our study, the mRNA level of NR1 subunit in the IO declined with maturation (as indicated by in situ hybridization) while the immunoreactivity did not follow the trend (as shown by immunohistochemistry). Indeed, such a mismatch between mRNA and protein expression level of NMDA receptor subunit has been observed in other brain regions. For example, during postnatal development of the visual cortex, the mRNA level of NR1 subunit declined dramatically between P22 and P45 (as indicated by in situ hybridization) but the protein level remained high (as indicated by immunoreactivity) (Cao et al. 2000). Disparities between mRNA and protein levels of the NR2A subunit were also shown in the developing visual cortex (Cao et al. 2000). Similarly, such disparities have also been reported in other neural regions (Gazzaley et al. 1996; Resink et al. 1995). It is therefore not uncommon that the receptor protein subunits in association with other partners in the postsynaptic membrane persist longer than the corresponding mRNAs.

In most neural areas, including the otolith-related IO neurons currently studied, developmental decrease in NR2B subunits is paralleled with the increase of NR2A subunits (Quinlan et al. 1999b; Ritter et al. 2002; Sheng et al. 1994; Stocca and Vicini 1998). In IO, unlike other neural areas where NR2B subunits are highly expressed in the first postnatal week (Akazawa et al. 1994; Monyer et al. 1994; Quinlan et al. 1999a; Shi et al. 1997; Watanabe et al. 1994), the highest level of NR2B subunit occurred during the second postnatal week. Since the postsynaptic currents mediated by NR2B-rich receptors are conducive to dynamic synaptic changes (Scheetz and Constantine-Paton 1994), the prolonged expression of NR2B subunits in otolith-related IO neurons might be important for synaptic organization during refinement of the olivo-cerebellar pathway around the end of the second postnatal week.

All Fos-ir neurons examined in the present study expressed GluR2 subunits which are non-permeable to Ca^2+^, suggesting that the operation of AMPA receptors in otolith-related neurons within the DMCC and IOβ is probably mediated by Ca^2+^-impermeable AMPA receptors. Even though the co-expression of NR1 and GluR2 subunits was not examined in the present study, the ubiquitous expression of both subunits in nearly all Fos-ir IO neurons tempts us to deduce that these subunits are co-localized to trigger the synergistic operation of the AMPA and NMDA receptors (Argence et al. 2006; Kharazia and Weinberg 1999) for processing spatial information during postnatal development.

We have therefore provided evidence that IO neurons that process gravity-related movements in the 3-D space express AMPA and NMDA receptors. Interestingly, developmental expression of glutamate receptor-mediated subthreshold oscillation of membrane potentials that are important in modulating the response dynamics of IO neurons (Llinás 2009) as well as the establishment of one-to-one olivo-cerebellar connection both occur by P14 (Crépel et al. 1976; Mariani and Changeux 1981). Our results therefore support that ionotropic glutamate receptors enable postnatal transmission of vestibular signals to IO neurons of distinct functional entities and also contribute to the remodeling of the olivo-cerebellar neural circuitry during maturation.

## Abbreviations

AMPA: α-Amino-3-hydroxy-5-methyl-4-isoxazolepropionate
cIOβ: Caudal IOβ
DAB: Diaminobenzidine
DMCC: Dorso-medial cell column of inferior olive
For-ir: Fos-immunoreactive
IO: Inferior olive
IOA: Subnucleus A of inferior olive
IOB: Subnucleus B of inferior olive
IOC: Subnucleus C of inferior olive
IOD: Dorsal accessory olive
IOK: Cap of Kooy inferior olive (dorsal cap)
IOM: Medial subnucleus of inferior olive
IOPr: Principal nucleus of inferior olive
IOV: Ventrolateral outgrowth of inferior olive
IOβ: Beta nucleus of inferior olive
LC: Locus coeruleus
NMDA: *N*-methyl-d-aspartate
P: Postnatal day
PBS: Phosphate buffered saline
rIOβ: Rostral IOβ
